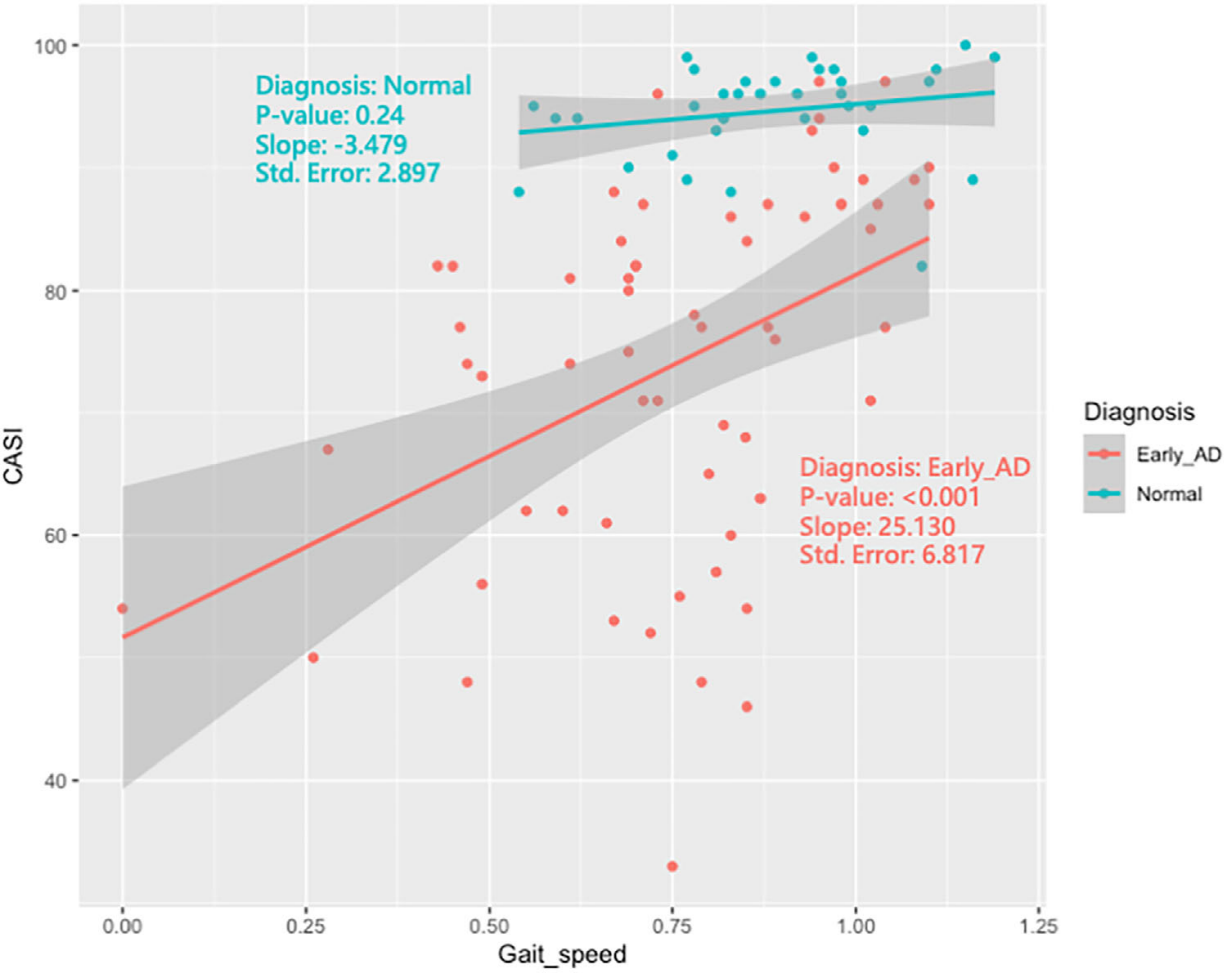# Gait Speed Reflects Cognitive Impairment in Early AD

**DOI:** 10.1002/alz.086732

**Published:** 2025-01-03

**Authors:** Che‐Sheng Wu, Chia‐Ju Chou, Chih‐Ting Chang, Chia‐Ying Lee, Yi‐ Fang Chuang, Yen‐Ling Chiu, Yi‐Chien Liu

**Affiliations:** ^1^ Medical school of Fu‐Jen University, New Taipei Taiwan; ^2^ Cardinal Tien Hospital, New Taipei City Taiwan; ^3^ National Taipei University of Nursing and Health Sciences, Taipei Taiwan; ^4^ Institute of Linguistics, Academia Sinica, Taipei Taiwan; ^5^ National Yang Ming Chiao Tung University, Taipei, NA Taiwan; ^6^ Far‐Eastern Memorial Hospital, New Taipei Taiwan; ^7^ Tohoku University, Sendai Japan; ^8^ Medical school of Fu‐Jen University, Taipei Taiwan

## Abstract

**Background:**

Gait performance has been found to be an effective method for screening cognitive impairment in elderly. Nevertheless, the efficacy of utilizing gait speed as a marker for monitoring cognitive changes remains incompletely substantiated.

**Method:**

From 2021 to 2023, we recruited 104 participants from the memory clinic of Cardinal Tien Hospital in Taipei, Taiwan. Individuals with organic brain lesions, joint diseases, or any other factors affecting gait speed were excluded. All participants underwent comprehensive neuropsychological tests and the Short Physical Performance Battery (SPPB) to assess physical function. The clinical diagnosis of Alzheimer disease (AD) was established using the 2021 NIA‐AA criteria. Global cognitive impairment was determined using the Cognitive Abilities Screening Instrument (CASI), and gait speed was measured through a 4‐meter walking test derived from the SPPB. Cognition in specific domains was assessed using various tests, such as the Logical Memory Test (LM) from the Wechsler Memory Scale 3rd edition, the Color Trail Making Test (CTT), and the Verbal Fluency Test (VF).

**Result:**

This study revealed a significant difference in SPPB score (11.03 vs. 12.64, p < 0.001) and gait speed (0.78 vs. 0.95 m/s, p = 0.005) between the early AD and normal groups. Furthermore, a positive correlation was observed between gait speed and CASI scores (r = 0.421, p < 0.001) in the early AD group, while no such effect was observed in the normal group (p = 0.24). In multiple regression analysis, our study demonstrated that CASI was better predicted by gait speed (p = 0.001) rather than age, gender, or years of education. LM (r = 0.393, p = 0.001), CTT (r = ‐0.449, p = 0.001), and VF (r = 0.349, p = 0.004) also revealed a significant correlation with gait speed in the early AD group.

**Conclusion:**

Gait speed positively correlated with cognitive impairment in the early AD, but not in normal controls. One possible explanation is the pathological changes of AD in the brain that affect gait and cognition simultaneously. Our study suggests that gait speed should be considered as a marker reflecting global cognitive function.